# Editorial Comment to Paraurethral Endometrioid Carcinoma Arising From Ectopic Endometriosis: A Case Report

**DOI:** 10.1002/iju5.70269

**Published:** 2026-09-20

**Authors:** Yosuke Tarumi, Taisuke Mori

**Affiliations:** ^1^ Department of Obstetrics and Gynecology Kyoto Prefectural University of Medicine, Graduate School of Medical Science Kyoto Japan

Endometriosis is defined as the presence of endometrium‐like tissue outside the uterus and commonly affects pelvic organs. However, endometriotic lesions could occur at rare sites, including the bowel, bladder, abdominal wall, diaphragm, lung, umbilicus, inguinal canal, and liver. Malignant transformation (MT) of endometriosis is rare, occurring in an estimated 0.5%–1.0% of cases, and endometriosis is associated with an increased risk of ovarian malignancy [1, 2]. Although MT of endometriosis at various rare sites has been reported, paraurethral endometriosis itself is extremely uncommon, and, to our knowledge, this is the first reported case of malignancy associated with paraurethral endometriosis [3].

The criteria for the diagnosis of malignancy arising from endometriosis as defined by Sampson and Scott is as follows: (1) the presence of both benign and neoplastic endometrial tissues in the tumor, (2) histological findings compatible with an endometrial origin, (3) the discovery of no other primary tumor sites, and (4) a morphologic demonstration of a continuum between benign and malignant epithelium [2]. However, such transition in (4) criteria is detectable in only 36%–42% of extraovarian endometriosis‐associated malignancies, possibly because of tumor overgrowth or limited sampling. More recently, endometriosis‐associated ovarian cancer (EAOC), fulfilling (1–3) criteria, has been distinguished from ovarian cancer arising in endometriosis (OCAE), which additionally fulfills (4) criteria [4]. Thus, absence of a demonstrable transition does not necessarily exclude an association with endometriosis. In the present case, although (4) criterion was not fulfilled, the tumor was considered to be associated with paraurethral endometriosis, additionally supported by the profile of PTEN and ARID1A alterations.

An unresolved issue for MT from rare site endometriosis is how these malignancies should be classified, staged, and treated. Although these tumors may share histogenesis and biological characteristics with endometriosis‐associated ovarian cancer as presented in this case, their clinical behavior could be influenced by the anatomical site of origin. Clear cell carcinomas arising from rectal and sigmoid endometriosis have been reported to metastasize to regional lymph node, such as pericolic and perisigmoid, respectively [5]. These findings suggest that lymphatic metastasis could follow the drainage pathways of the organ in which MT occurs. Therefore, staging based solely on ovarian or peritoneal cancer classification may not fully reflect the anatomical pattern of tumor spread. Similarly, whether systemic treatment, particularly surgical resection, should follow ovarian cancer protocols or organ‐specific strategies remains unclear. Further accumulation of cases is required to establish appropriate classification, staging, and treatment strategies for these rare malignancies.

## Conflicts of Interest

The authors declare no conflicts of interest.

## Data Availability

Data sharing not applicable to this article as no datasets were generated or analysed during the current study.
